# 2,2-Dimethyl-5-[(2-nitro­anilino)methyl­idene]-1,3-dioxane-4,6-dione

**DOI:** 10.1107/S1600536811014358

**Published:** 2011-04-29

**Authors:** Yu-xin He, Jin-wei Wu, Rong-sheng Tong, Jian-you Shi

**Affiliations:** a Bioengineering College, Xihua University, Chengdu, Sichuan 610039, People’s Republic of China; b Sichuan Academy of Medical Sciences and Sichuan Provincial People’s Hospital, Chengdu, Sichuan 610072, People’s Republic of China

## Abstract

The crystal of the title compound, C_13_H_12_N_2_O_6_, contains a bifurcated intra­molecular hydrogen bond between the N—H group and one of the O atoms from both the nitro group and the dioxane-4,6-dione moiety. In addition, mol­ecules are linked by a series of inter­molecular C—H⋯O secondary inter­actions. The dihedral angles between the benzene ring and the nitro group and the conjugated part of the dioxane-4,6-dione moiety are 19.1 (2) and 17.89 (7)°, respectively.

## Related literature

The title compound is an important intermediate drug discovery. For the synthesis and structures of related antitumor precursors, see: Cassis *et al.* (1985 ▶). For related literature, see Dolomanov *et al.* (2009 ▶).
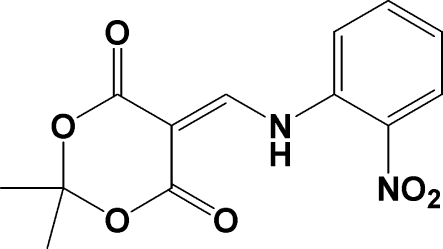


## Experimental

### 

#### Crystal data

C_13_H_12_N_2_O_6_
*M* *_r_* = 292.25
Monoclinic, 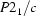

*a* = 6.3860 (2) Å
*b* = 17.3800 (5) Å
*c* = 11.9338 (3) Å
β = 90.622 (3)°
*V* = 1324.44 (7) Å^3^
*Z* = 4
Mo *K*α radiation
μ = 0.12 mm^−1^
*T* = 150 K
0.42 × 0.35 × 0.25 mm

#### Data collection

Oxford Diffraction Xcalibur Eos diffractometer
Absorption correction: multi-scan (*CrysAlis PRO*; Oxford Diffraction, 2010 ▶) *T*_min_ = 0.993, *T*_max_ = 1.0
9157 measured reflections
2693 independent reflections
2212 reflections with *I* > 2σ(*I*)
*R*_int_ = 0.027

#### Refinement

*R*[*F* ^2^ > 2σ(*F* ^2^)] = 0.038
*wR*(*F* ^2^) = 0.097
*S* = 1.03
2693 reflections
192 parameters
H-atom parameters constrained
Δρ_max_ = 0.21 e Å^−3^
Δρ_min_ = −0.22 e Å^−3^

### 

Data collection: *CrysAlis PRO* (Oxford Diffraction, 2010 ▶); cell refinement: *CrysAlis PRO*; data reduction: *CrysAlis PRO*; program(s) used to solve structure: *SHELXS97* (Sheldrick, 2008 ▶); program(s) used to refine structure: *SHELXL97* (Sheldrick, 2008 ▶); molecular graphics: *OLEX2* (Dolomanov *et al.*, 2009 ▶); software used to prepare material for publication: *OLEX2*.

## Supplementary Material

Crystal structure: contains datablocks global, I. DOI: 10.1107/S1600536811014358/bv2184sup1.cif

Structure factors: contains datablocks I. DOI: 10.1107/S1600536811014358/bv2184Isup2.hkl

Additional supplementary materials:  crystallographic information; 3D view; checkCIF report

## Figures and Tables

**Table 1:** Hydrogen-bond geometry (Å, °)

*D*—H⋯*A*	*D*—H	H⋯*A*	*D*⋯*A*	*D*—H⋯*A*
N1—H1⋯O5	0.88	1.97	2.6403 (16)	132
N1—H1⋯O3	0.88	2.10	2.7439 (16)	130
C7—H7⋯O4^i^	0.95	2.40	3.0852 (18)	129
C10—H10⋯O6^ii^	0.95	2.48	3.4219 (19)	170
C11—H11⋯O1^iii^	0.95	2.53	3.4508 (18)	162
C13—H13⋯O4^i^	0.95	2.53	3.4445 (18)	161

Symmetry codes: (i) 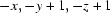; (ii) 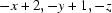; (iii) 
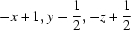
.

**Fig. 1.**
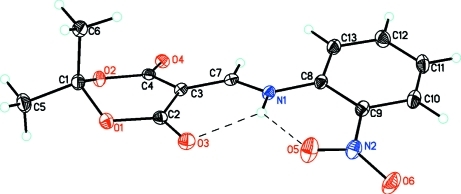
The molecular structure of the title compound showing the bifurcated intramolecular hydrogen bond.

**Fig. 2.**
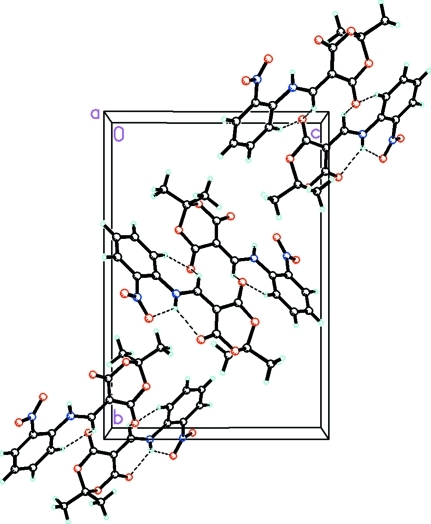
Fi. 2. The packing diagram for the title compound viewed down the a axis, showing the intermolecular C—H···O interactions.

## supplementary crystallographic information

### Comment

2,2-Dimethyl-5-[(2-nitrophenylamino)-methylene]-[1,3]dioxane-4,6-dione, C_13_H_12_N_2_O_6_, is a key intermediate which can be used to synthesize the 4(1*H*)quinolone derivatives by thermolysis, which can then be used as precursors for anti-malarial agents or anti-cancer agents. The structure contains an bifurcated intramolecular hydrogen bond between the N-H and one of the O's from both the nitro group and the dioxane-4,6-dione moiety. In addition the molecules are linked by a series of intermolecular C-H···O secondary interactions. The dihedral angles between the phenyl group and both the nitro and conjugated part of the dioxane-4,6-dione moiety are 19.1 (2)° and 17.89 (7)°, respectively.

### Experimental

A mixture of 2,2-dimethyl-1,3-dioxane-4,6-dione(1.44 g, 0.01 mol) and methylorthoformate (1.27 g, 0.012 mol) was heated to reflux for 0.5 h, then 2-nitroaniline(1.38 g, 0.01 mol) in ethanol (20 mL) was added into the above solution. The mixture was heated under reflux for another 2 h and poured into cold water then filtered to obtain a powder. Single crystals were obtained from the powder in CH_2_Cl_2_ and methanol after 3 days.

### Refinement

H atoms were positioned geometrically (C—H = 0.93–0.98 Å) and refined using a riding model, with *U*_iso_(H) = 1.2*U*_eq_(C) [*U*_iso_(H) = 1.5*U*_eq_(C) for the CH_3_ groups).

### Crystal data

**Table:**

C_13_H_12_N_2_O_6_	*F*(000) = 608
*M**_r_* = 292.25	*D*_x_ = 1.466 Mg m^−^^3^
Monoclinic, *P*2_1_/*c*	Mo *K*α radiation, λ = 0.71070 Å
Hall symbol: -P 2ybc	Cell parameters from 3956 reflections
*a* = 6.3860 (2) Å	θ = 2.9–29.1°
*b* = 17.3800 (5) Å	µ = 0.12 mm^−^^1^
*c* = 11.9338 (3) Å	*T* = 150 K
β = 90.622 (3)°	Block, colourless
*V* = 1324.44 (7) Å^3^	0.42 × 0.35 × 0.25 mm
*Z* = 4	

### Data collection

**Table:**

Oxford Diffraction Xcalibur Eos diffractometer	2693 independent reflections
Radiation source: fine-focus sealed tube	2212 reflections with *I* > 2σ(*I*)
graphite	*R*_int_ = 0.027
Detector resolution: 16.0874 pixels mm^-1^	θ_max_ = 26.4°, θ_min_ = 2.9°
ω scans	*h* = −7→7
Absorption correction: multi-scan (*CrysAlis PRO*; Oxford Diffraction, 2010)	*k* = 0→21
*T*_min_ = 0.993, *T*_max_ = 1.0	*l* = 0→14
9157 measured reflections	

### Refinement

**Table:**

Refinement on *F*^2^	Primary atom site location: structure-invariant direct methods
Least-squares matrix: full	Secondary atom site location: difference Fourier map
*R*[*F*^2^ > 2σ(*F*^2^)] = 0.038	Hydrogen site location: inferred from neighbouring sites
*wR*(*F*^2^) = 0.097	H-atom parameters constrained
*S* = 1.03	*w* = 1/[σ^2^(*F*_o_^2^) + (0.0439*P*)^2^ + 0.3082*P*] where *P* = (*F*_o_^2^ + 2*F*_c_^2^)/3
2693 reflections	(Δ/σ)_max_ < 0.001
192 parameters	Δρ_max_ = 0.21 e Å^−^^3^
0 restraints	Δρ_min_ = −0.22 e Å^−^^3^

### Special details

**Table:**

Geometry. All esds (except the esd in the dihedral angle between two l.s. planes) are estimated using the full covariance matrix. The cell esds are taken into account individually in the estimation of esds in distances, angles and torsion angles; correlations between esds in cell parameters are only used when they are defined by crystal symmetry. An approximate (isotropic) treatment of cell esds is used for estimating esds involving l.s. planes.
Refinement. Refinement of F^2^ against ALL reflections. The weighted R-factor wR and goodness of fit S are based on F^2^, conventional R-factors R are based on F, with F set to zero for negative F^2^. The threshold expression of F^2^ > 2sigma(F^2^) is used only for calculating R-factors(gt) etc. and is not relevant to the choice of reflections for refinement. R-factors based on F^2^ are statistically about twice as large as those based on F, and R- factors based on ALL data will be even larger.

### Fractional atomic coordinates and isotropic or equivalent isotropic displacement parameters (Å^2^)

**Table:**

	*x*	*y*	*z*	*U*_iso_*/*U*_eq_	
O1	0.38713 (16)	0.72407 (6)	0.58099 (8)	0.0262 (3)	
O2	0.14846 (17)	0.64025 (6)	0.66934 (8)	0.0260 (3)	
O3	0.57240 (17)	0.68777 (6)	0.43461 (9)	0.0287 (3)	
O4	0.10877 (16)	0.51978 (6)	0.61477 (8)	0.0255 (3)	
O5	0.7590 (2)	0.62314 (7)	0.21713 (11)	0.0434 (3)	
O6	0.9663 (2)	0.55108 (8)	0.12201 (10)	0.0452 (3)	
N1	0.45628 (19)	0.55433 (7)	0.32793 (9)	0.0215 (3)	
H1	0.5390	0.5948	0.3241	0.026*	
N2	0.7992 (2)	0.56246 (8)	0.16919 (10)	0.0302 (3)	
C1	0.1846 (2)	0.71834 (8)	0.63308 (12)	0.0254 (3)	
C2	0.4337 (2)	0.67221 (8)	0.49964 (11)	0.0218 (3)	
C3	0.3173 (2)	0.60086 (8)	0.50100 (11)	0.0204 (3)	
C4	0.1833 (2)	0.58240 (8)	0.59513 (11)	0.0211 (3)	
C5	0.1985 (3)	0.76619 (10)	0.73797 (13)	0.0358 (4)	
H5A	0.3132	0.7472	0.7856	0.054*	
H5B	0.0664	0.7626	0.7787	0.054*	
H5C	0.2249	0.8200	0.7180	0.054*	
C6	0.0136 (3)	0.74320 (10)	0.55292 (13)	0.0329 (4)	
H6A	0.0098	0.7084	0.4884	0.049*	
H6B	0.0413	0.7958	0.5273	0.049*	
H6C	−0.1215	0.7417	0.5910	0.049*	
C7	0.3356 (2)	0.54685 (8)	0.41734 (12)	0.0204 (3)	
H7	0.2563	0.5009	0.4240	0.025*	
C8	0.4737 (2)	0.50006 (8)	0.24088 (12)	0.0216 (3)	
C9	0.6411 (2)	0.50180 (8)	0.16525 (12)	0.0238 (3)	
C10	0.6632 (3)	0.44616 (9)	0.08294 (12)	0.0295 (4)	
H10	0.7784	0.4482	0.0333	0.035*	
C11	0.5182 (3)	0.38812 (9)	0.07327 (13)	0.0323 (4)	
H11	0.5328	0.3498	0.0171	0.039*	
C12	0.3506 (3)	0.38574 (9)	0.14598 (14)	0.0334 (4)	
H12	0.2503	0.3456	0.1394	0.040*	
C13	0.3277 (2)	0.44111 (9)	0.22799 (13)	0.0283 (4)	
H13	0.2105	0.4390	0.2763	0.034*	

### Atomic displacement parameters (Å^2^)

**Table:**

	*U*^11^	*U*^22^	*U*^33^	*U*^12^	*U*^13^	*U*^23^
O1	0.0282 (6)	0.0224 (5)	0.0279 (6)	−0.0010 (4)	−0.0001 (5)	−0.0038 (4)
O2	0.0299 (6)	0.0280 (6)	0.0201 (5)	0.0010 (5)	0.0039 (4)	−0.0033 (4)
O3	0.0266 (6)	0.0276 (6)	0.0322 (6)	−0.0059 (5)	0.0055 (5)	0.0002 (5)
O4	0.0235 (6)	0.0290 (6)	0.0239 (5)	−0.0056 (4)	0.0029 (4)	0.0009 (4)
O5	0.0497 (8)	0.0287 (6)	0.0524 (8)	−0.0103 (6)	0.0263 (6)	−0.0056 (6)
O6	0.0343 (7)	0.0534 (8)	0.0484 (8)	−0.0045 (6)	0.0235 (6)	−0.0030 (6)
N1	0.0198 (7)	0.0203 (6)	0.0245 (6)	−0.0010 (5)	0.0048 (5)	−0.0014 (5)
N2	0.0316 (8)	0.0324 (7)	0.0269 (7)	−0.0017 (6)	0.0115 (6)	0.0042 (6)
C1	0.0283 (8)	0.0248 (8)	0.0230 (7)	0.0031 (7)	0.0007 (6)	−0.0027 (6)
C2	0.0213 (8)	0.0227 (7)	0.0213 (7)	0.0017 (6)	−0.0027 (6)	0.0006 (6)
C3	0.0178 (7)	0.0214 (7)	0.0219 (7)	0.0012 (6)	0.0014 (6)	0.0000 (6)
C4	0.0172 (7)	0.0268 (8)	0.0193 (7)	0.0002 (6)	−0.0024 (6)	−0.0004 (6)
C5	0.0463 (11)	0.0336 (9)	0.0275 (8)	0.0052 (8)	−0.0038 (8)	−0.0085 (7)
C6	0.0332 (10)	0.0365 (9)	0.0289 (8)	0.0100 (7)	−0.0046 (7)	−0.0062 (7)
C7	0.0159 (7)	0.0209 (7)	0.0245 (7)	0.0001 (6)	0.0000 (6)	0.0024 (6)
C8	0.0226 (8)	0.0208 (7)	0.0214 (7)	0.0045 (6)	0.0015 (6)	0.0007 (6)
C9	0.0250 (8)	0.0245 (7)	0.0219 (7)	0.0029 (6)	0.0044 (6)	0.0039 (6)
C10	0.0344 (9)	0.0317 (9)	0.0227 (8)	0.0095 (7)	0.0068 (7)	0.0028 (6)
C11	0.0435 (10)	0.0284 (8)	0.0251 (8)	0.0078 (7)	0.0017 (7)	−0.0064 (6)
C12	0.0368 (10)	0.0286 (8)	0.0347 (9)	−0.0043 (7)	0.0009 (7)	−0.0065 (7)
C13	0.0247 (9)	0.0310 (8)	0.0294 (8)	−0.0021 (7)	0.0055 (7)	−0.0044 (6)

### Geometric parameters (Å, °)

**Table:**

O1—C2	1.3600 (17)	C5—H5A	0.9800
O1—C1	1.4446 (17)	C5—H5B	0.9800
O2—C4	1.3598 (17)	C5—H5C	0.9800
O2—C1	1.4438 (17)	C6—H6A	0.9800
O3—C2	1.2144 (17)	C6—H6B	0.9800
O4—C4	1.2118 (17)	C6—H6C	0.9800
O5—N2	1.2283 (17)	C7—H7	0.9500
O6—N2	1.2276 (17)	C8—C13	1.393 (2)
N1—C7	1.3292 (18)	C8—C9	1.4066 (19)
N1—C8	1.4084 (18)	C9—C10	1.387 (2)
N1—H1	0.8808	C10—C11	1.373 (2)
N2—C9	1.460 (2)	C10—H10	0.9500
C1—C5	1.505 (2)	C11—C12	1.386 (2)
C1—C6	1.507 (2)	C11—H11	0.9500
C2—C3	1.446 (2)	C12—C13	1.382 (2)
C3—C7	1.376 (2)	C12—H12	0.9500
C3—C4	1.4551 (19)	C13—H13	0.9500
			
C2—O1—C1	117.73 (11)	H5B—C5—H5C	109.5
C4—O2—C1	118.16 (10)	C1—C6—H6A	109.5
C7—N1—C8	125.29 (12)	C1—C6—H6B	109.5
C7—N1—H1	118.2	H6A—C6—H6B	109.5
C8—N1—H1	116.4	C1—C6—H6C	109.5
O6—N2—O5	122.62 (14)	H6A—C6—H6C	109.5
O6—N2—C9	118.24 (13)	H6B—C6—H6C	109.5
O5—N2—C9	119.12 (12)	N1—C7—C3	124.77 (14)
O2—C1—O1	109.90 (11)	N1—C7—H7	117.6
O2—C1—C5	106.16 (12)	C3—C7—H7	117.6
O1—C1—C5	105.96 (13)	C13—C8—C9	117.22 (13)
O2—C1—C6	110.05 (13)	C13—C8—N1	121.06 (13)
O1—C1—C6	110.67 (12)	C9—C8—N1	121.70 (13)
C5—C1—C6	113.91 (13)	C10—C9—C8	121.56 (14)
O3—C2—O1	118.37 (13)	C10—C9—N2	116.77 (13)
O3—C2—C3	125.22 (13)	C8—C9—N2	121.67 (13)
O1—C2—C3	116.35 (12)	C11—C10—C9	119.87 (15)
C7—C3—C2	121.94 (13)	C11—C10—H10	120.1
C7—C3—C4	117.71 (13)	C9—C10—H10	120.1
C2—C3—C4	120.28 (12)	C10—C11—C12	119.61 (14)
O4—C4—O2	118.07 (12)	C10—C11—H11	120.2
O4—C4—C3	125.65 (13)	C12—C11—H11	120.2
O2—C4—C3	116.21 (12)	C13—C12—C11	120.75 (15)
C1—C5—H5A	109.5	C13—C12—H12	119.6
C1—C5—H5B	109.5	C11—C12—H12	119.6
H5A—C5—H5B	109.5	C12—C13—C8	120.97 (14)
C1—C5—H5C	109.5	C12—C13—H13	119.5
H5A—C5—H5C	109.5	C8—C13—H13	119.5
			
C4—O2—C1—O1	−49.01 (16)	C2—C3—C7—N1	−0.9 (2)
C4—O2—C1—C5	−163.17 (13)	C4—C3—C7—N1	−177.98 (13)
C4—O2—C1—C6	73.12 (16)	C7—N1—C8—C13	15.3 (2)
C2—O1—C1—O2	50.40 (15)	C7—N1—C8—C9	−163.14 (14)
C2—O1—C1—C5	164.70 (12)	C13—C8—C9—C10	−1.6 (2)
C2—O1—C1—C6	−71.36 (15)	N1—C8—C9—C10	176.90 (14)
C1—O1—C2—O3	160.00 (13)	C13—C8—C9—N2	178.09 (14)
C1—O1—C2—C3	−22.41 (18)	N1—C8—C9—N2	−3.4 (2)
O3—C2—C3—C7	−8.8 (2)	O6—N2—C9—C10	−18.5 (2)
O1—C2—C3—C7	173.82 (13)	O5—N2—C9—C10	159.99 (15)
O3—C2—C3—C4	168.19 (14)	O6—N2—C9—C8	161.84 (14)
O1—C2—C3—C4	−9.2 (2)	O5—N2—C9—C8	−19.7 (2)
C1—O2—C4—O4	−163.13 (13)	C8—C9—C10—C11	0.7 (2)
C1—O2—C4—C3	19.81 (18)	N2—C9—C10—C11	−178.97 (14)
C7—C3—C4—O4	10.9 (2)	C9—C10—C11—C12	0.1 (2)
C2—C3—C4—O4	−166.25 (14)	C10—C11—C12—C13	0.0 (2)
C7—C3—C4—O2	−172.34 (13)	C11—C12—C13—C8	−1.0 (3)
C2—C3—C4—O2	10.6 (2)	C9—C8—C13—C12	1.7 (2)
C8—N1—C7—C3	−178.89 (14)	N1—C8—C13—C12	−176.80 (14)

### Hydrogen-bond geometry (Å, °)

**Table:**

*D*—H···*A*	*D*—H	H···*A*	*D*···*A*	*D*—H···*A*
N1—H1···O5	0.88	1.97	2.6403 (16)	132.
N1—H1···O3	0.88	2.10	2.7439 (16)	130.
C7—H7···O4^i^	0.95	2.40	3.0852 (18)	129.
C10—H10···O6^ii^	0.95	2.48	3.4219 (19)	170.
C11—H11···O1^iii^	0.95	2.53	3.4508 (18)	162.
C13—H13···O4^i^	0.95	2.53	3.4445 (18)	161.

Symmetry codes: (i) −*x*, −*y*+1, −*z*+1; (ii) −*x*+2, −*y*+1, −*z*; (iii) −*x*+1, *y*−1/2, −*z*+1/2.
